# Bill Nye and the Nurses

**Published:** 1899-08

**Authors:** 


### Bill Nye and the Nurses.

I have just been sent to the hospital for twenty days. My physician did it. He did it with an analysis. Anybody who amounts to anything nowadays gets analyzed. Sometimes you find casts, sometimes you find maple sugar, and sometimes you find acids, oxides, paint, oil, varnish, white lead, borax, albumin, lime, hair, and cement. In these cases the patient should be placed under a strict diet, or he will in the course of his life become a corpse. I go into details about this, because a false impression got out a few weeks ago to the effect that I came here for another purpose. A reporter came to see me, and I sent word to him that I was then out on the operating table in such a position that I could see no one, while an elderly surgeon was engaged in removing a porous plaster received during the war. I was not serious in saying this, but unfortunately I have the reputation for absolute veracity and seriousness, so that the statement got into the papers as bona fide, and caused American securities to go down two points in one day.—*Exchange.*

